# Supplementary material for: Myosin efflux promotes cell elongation to coordinate chromosome segregation with cell cleavage
Source: Nat Commun. 2017 Aug 23;8:326. doi: 10.1038/s41467-017-00337-6 (PMC5569077; doi:10.1038/s41467-017-00337-6)
Supplement: Supplementary file 6 — Supplementary Information [file 41467_2017_337_MOESM6_ESM.pdf]

Title: Supplementary Information

Description: Supplementary Figures and Supplementary Table

Title: Supplementary Movie 1

Description: **Myosin dynamics during the segregation of normal chromatids or trailing chromatids.** Time-lapse video of wild type neuroblasts with normal (left) or trailing chromatids (right) expressing H2Az::mRFP (red) and Sqh::GFP (grey). Images are maximum projections. Time= min:sec. Time 0:00 corresponds to anaphase onset (initiation of sister chromatid separation). The movie corresponds to figure 1a.

Title: Supplementary Movie 2

Description: **Myosin undergoes outward flux from the contractile ring towards the polar cortex.** Time-lapse video of a wild type neuroblast with normal chromatids expressing Sqh::GFP. Images correspond to one sagittal view of a cell that had already entered anaphase. Time= min:sec. The movie corresponds to figure 3a.

Title: Supplementary Movie 3

Description: **Myosin dynamics in pbl mutant cells during cytokinesis.** Time-lapse video of a pbl mutant cell with trailing chromatids expressing H2Az::mRFP and Sqh::GFP. Left images are maximum projection of H2Az::mRFP (red) and Sqh::GFP signal (grey). Right images are DIC. The movie corresponds to figure 4a, bottom row.

Title: Supplementary Movie 4

Description: **Nuclear envelope reassembly is delayed in the presence of trailing chromatids.** Time-lapse video of wild type neuroblasts with normal (left column) or trailing chromatids (right column) expressing H2Az::mRFP and GFP::Nup107. Top images are maximum projections of H2Az::mRFP (red) and GFP::Nup107 (grey). Bottom images are DIC. Time= min:sec. Time 0:00 corresponds to anaphase onset. The movie corresponds to figure 5a.

Title: Supplementary Movie 5

Description: **Myosin dynamics in pbl mutant cells expressing Pbl-NLSmut during cytokinesis.** Time-lapse video of a pbl mutant neuroblast with normal chromatids expressing expressing Pbl-NLSmut, H2Az::mRFP and Sqh::GFP. Left images are maximum projections of H2Az::RFP (red) and Sqh::GFP (grey). Right images are DIC. Time= min:sec. The movie corresponds to figure 6a (bottom row).

Title: Peer Review File

Description:

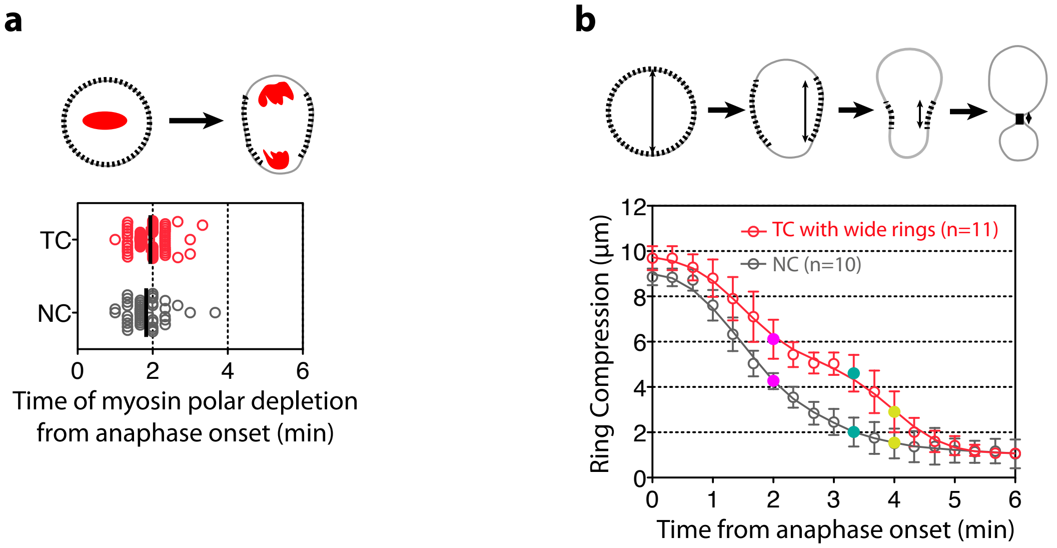

Supplementary figure 1 | The presence of trailing chromatids affects the rate of ring compression. (**a**) Scatter dot plot showing the time of myosin polar depletion from anaphase onset in cells with NC (n=40) and TC (n=77) as illustrated by the drawing above (chromatin mass in red, myosin distribution in black dashed curves). No difference is observed in the timing of myosin polar depletion between cells with NC and TC. (**b**) Graph showing the length of the contractile ring (ring compression) over time for cells with NC and cells with TC exhibiting wide rings at onset of furrowing. The scheme above the graph illustrates the method for measuring the length of the ring over time. The color-coded dots represent the average time of myosin depletion from both poles (purple), onset of furrowing (green) and initiation of efflux (yellow). Time starts at anaphase onset. The mean±CI95% is presented for the scatter dot plots and graphs.

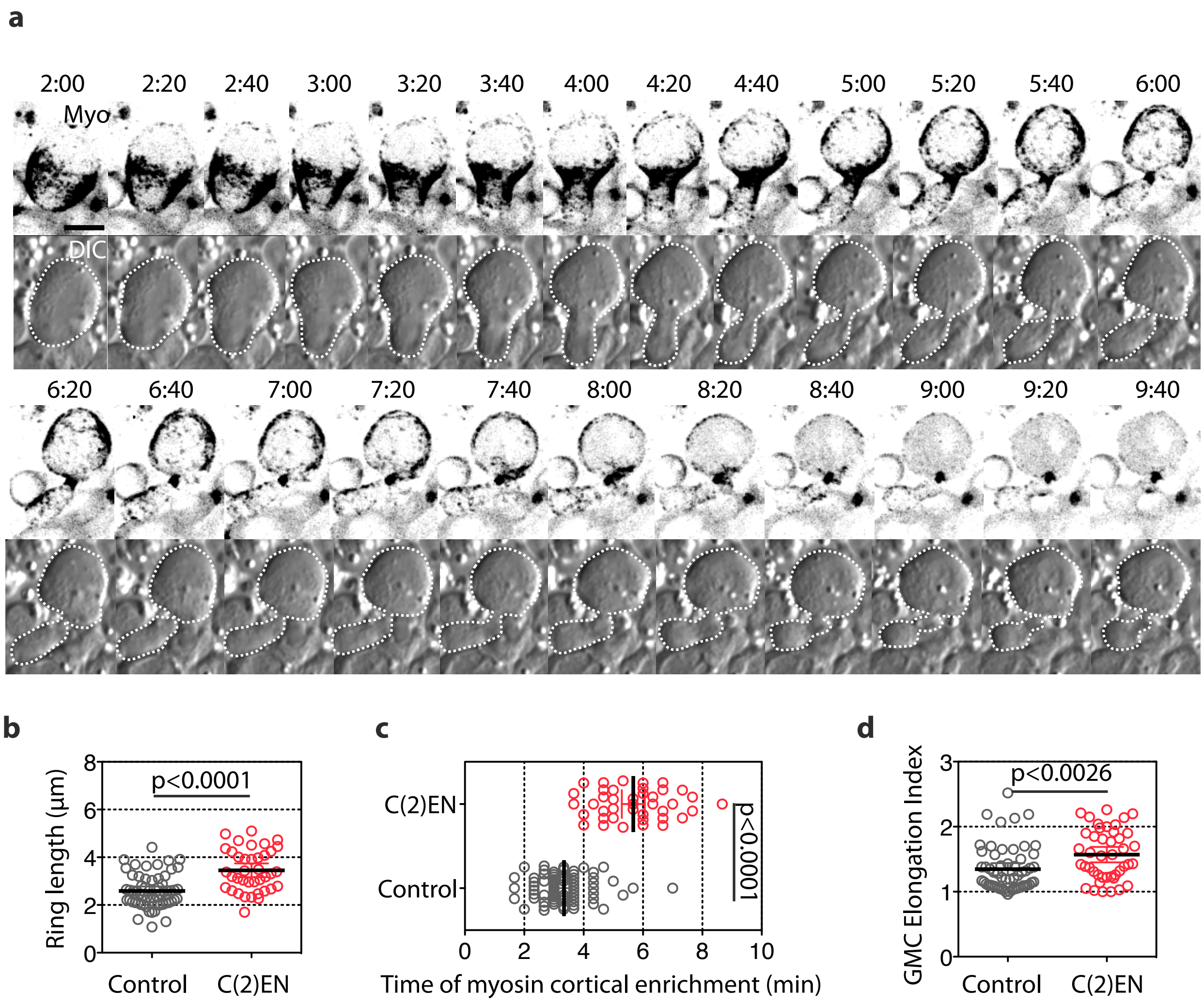

Supplementary figure 2 | Cells carrying C(2)EN exhibit prolonged myosin enrichment at the cortex. (**a**) Time-lapse images of a neuroblast carrying C(2)EN and expressing Sqh::GFP (Myo). Top panels are inverted images of Sqh::GFP and bottom panel are DIC images. Scale bar= 5µm. The white dashed lines outline the cell. Time=min: sec. Time starts at anaphase onset. (**b**) Scatter dot plot showing the distribution of the ring length at the onset of furrowing, as shown in fig. 1b and c. Cells carrying a C(2)EN assemble a wider contractile ring (NC, n=58; C(2)EN, n=38). (**c**) Scatter dot plot showing the duration of myosin cortical enrichment from initiation of efflux to disappearance. Myosin persists at the cortex for a longer time in cells carrying C(2)EN (NC, n=69; C(2)EN, n=40). (**d**) Scatter dot plot showing the distribution of the ring length at the onset of furrowing in WT (as shown in fig. 1d). Scatter dot plot showing the elongation index of cells with NC and cells carrying C(2)EN as described in fig. 2g and h (NC, n=57; C(2)EN, n=42). The mean±CI95% is presented for all scatter dot plots and graphs. A Mann-Whitney test was used to calculate P values.

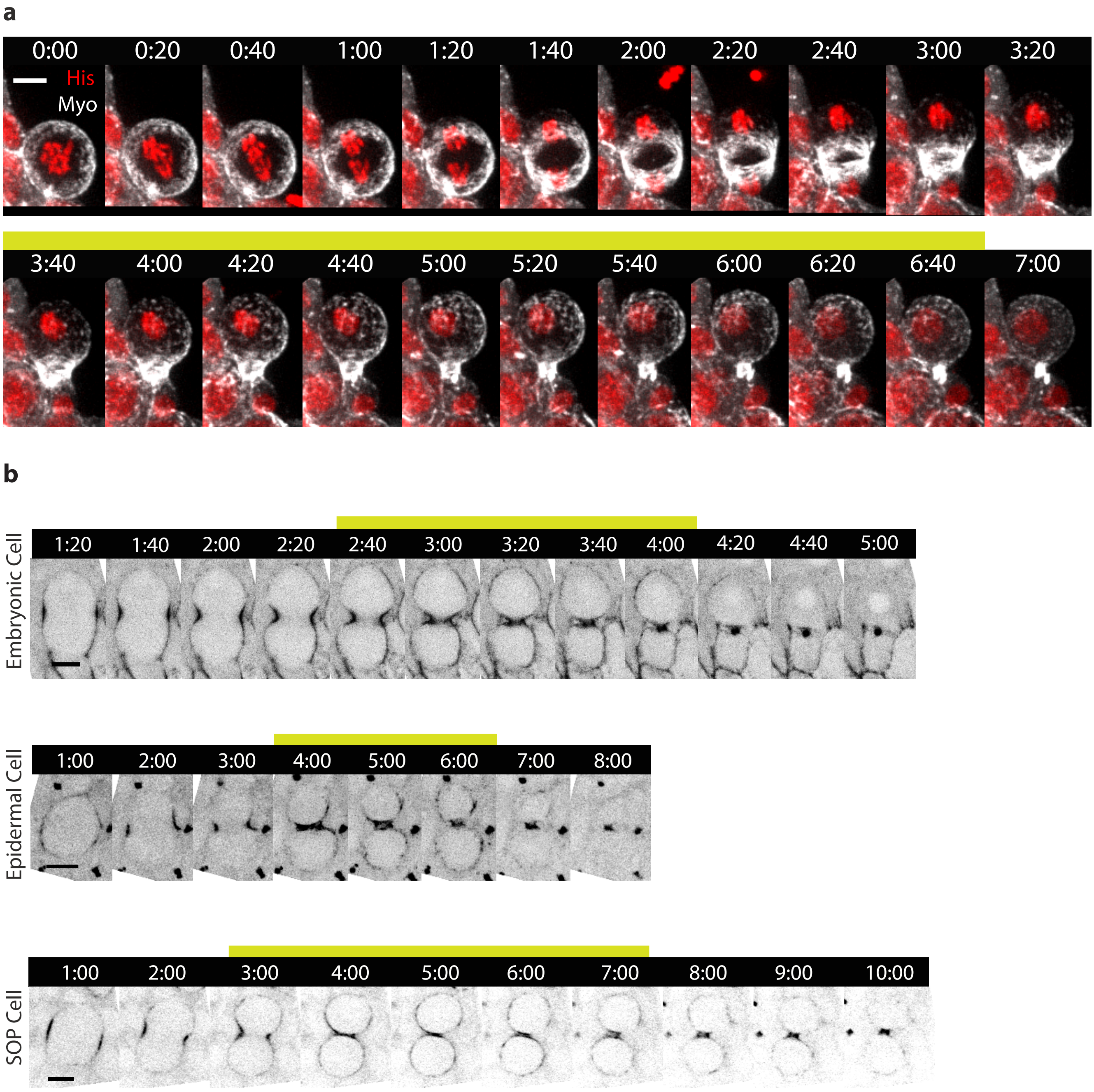

Supplementary figure 3 | Different types of Drosophila cells exhibit myosin efflux during cytokinesis. (**a**) Time-lapse images of *sqh[AX3]* mutant neuroblasts expressing Sqh::GFP (Myo, grey) and H2Az::mRFP (His, red). Myosin efflux is observed in *sqh* null mutant cells expressing one copy of Sqh::GFP. (**b**) Time-lapse images of a stage 15 embryonic cell (top panel), and two pupal epithelial cells (one epidermal cell (middle panel) and one sensory organ precursor pI cell (bottom panel)) expressing Sqh::GFP. The yellow bars above the images show the duration of myosin efflux from initiation to disappearance. Time 0:00 corresponds to anaphase onset. Time=min:sec. Scale bars= 5μm.

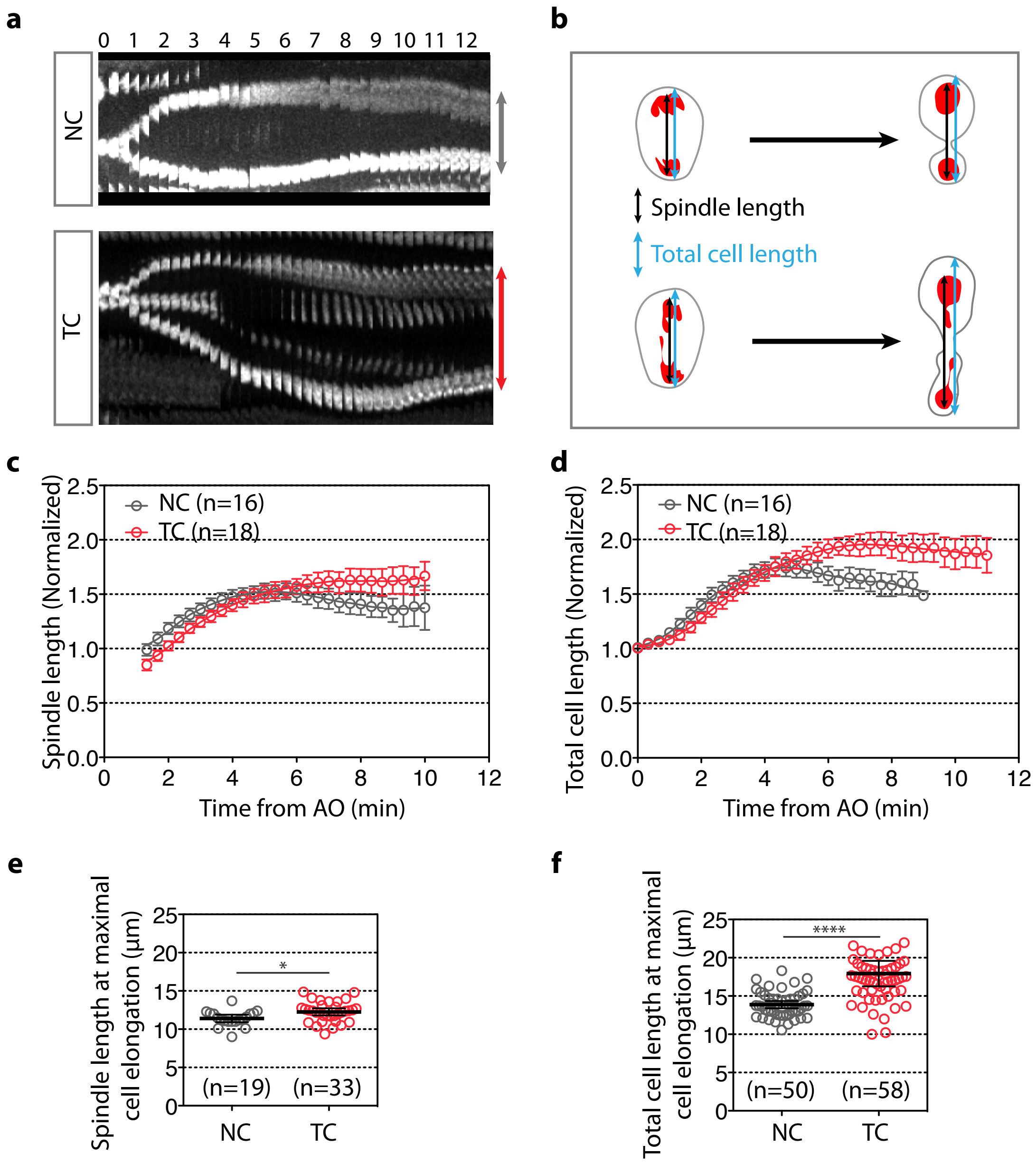

Supplementary figure 4 | Spindle length is only midly affected in the presence of trailing chromatids. (**a**) Kymographs of H2Az::mRFP signal in cells with NC or TC from one pole to the other over time. A straight line of 10 pixels was drawn from one pole to the other and plotted over time. Grey and red double-arrows correspond to the distance between the two chromatid masses (which correspond to spindle length) plotted in c for NC and TC cells respectively. Time 0 corresponds to anaphase onset. Time=min. (**b**) Drawings illustrating the method for measuring spindle length (black double-arrows) and total cell length (blue double-arrows) in cells with NC (top) and TC (bottom). (**c-d**) Graphs showing spindle length (c) and total cell length (d) over time in cells with NC and TC from anaphase onset (AO, time 0). The measurements are normalized with the diameter of the cell at metaphase. (**e-f**) Scatter dot plots showing the distribution of spindle length (e) and total cell length (f) in cells with NC and TC at the time after anaphase onset when they have reached their maximal elongation. n= number of cells. The mean±CI95% is presented for all graphs and scatter dot plots. A Mann-Whitney test was used to calculate P values (* corresponds to $P<0.05$, **** corresponds to $P<0.0001$).

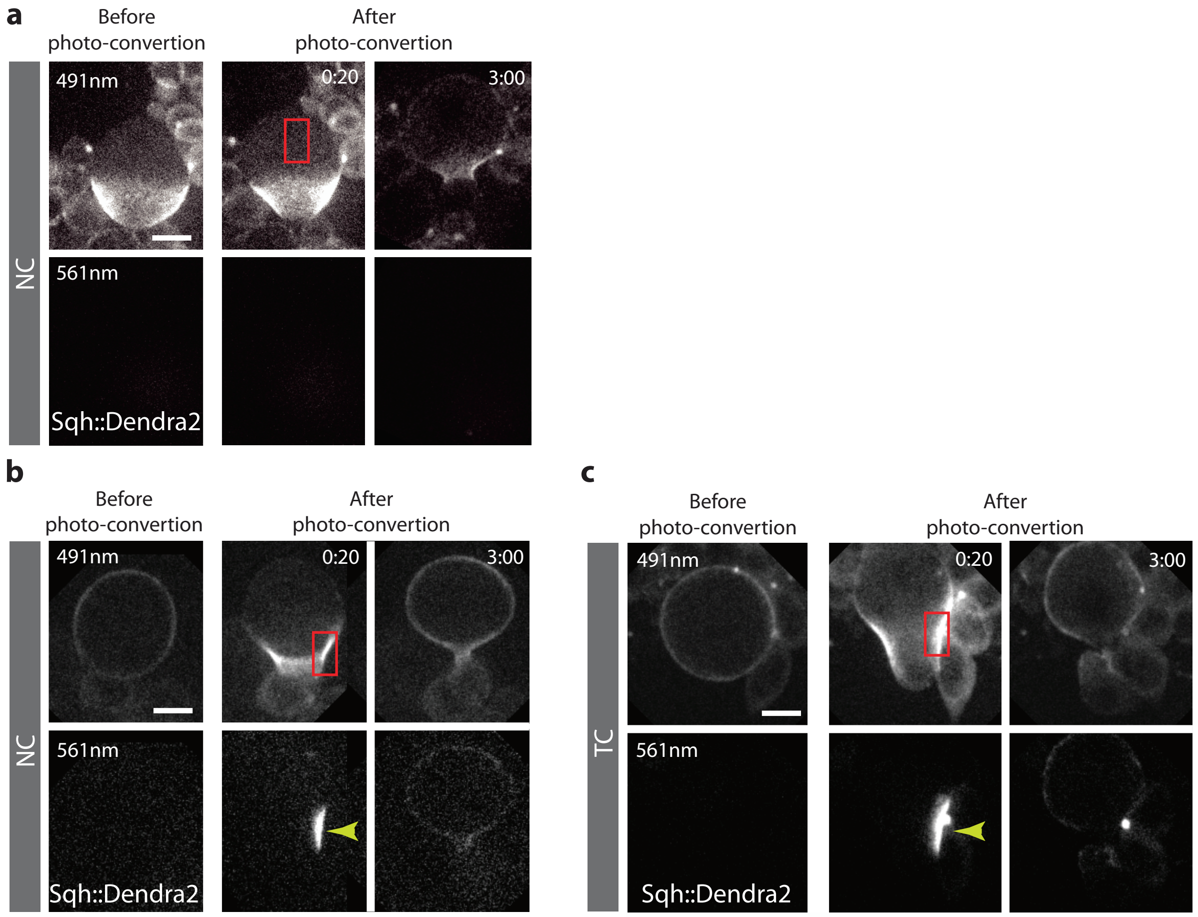

Supplementary figure 5 | Photo-converted myosin at the contractile ring is detected at the cortex of nascent daughter cells. (**a-c**) Images of live neuroblasts expressing Sqh::Dendra2. Dendra2 undergoes green to red photo-conversion upon absorption of 405nm light. No signal is detected with a 561nm excitation wavelength before photo-conversion. The red rectangle corresponds to the area photo-converted with the 405nm laser. (**a**) No signal is detected with the 561nm laser upon irradiation in the cytoplasm (time 0:00). (**b, c**) Upon irradiation at the site of the contractile ring (time 0:00), the pool of photo-converted Sqh::Dendra2 is rapidly visible with the 561nm laser (green arrowheads). Three minutes after irradiation, the pool of photo-converted Sqh::Dendra2 is detected around the whole cortex in cells with NC (b) and TC (c) similarly to the Sqh::Dendra2 detected with the 491nm laser. Time= min:sec. Scale bars=5μm.

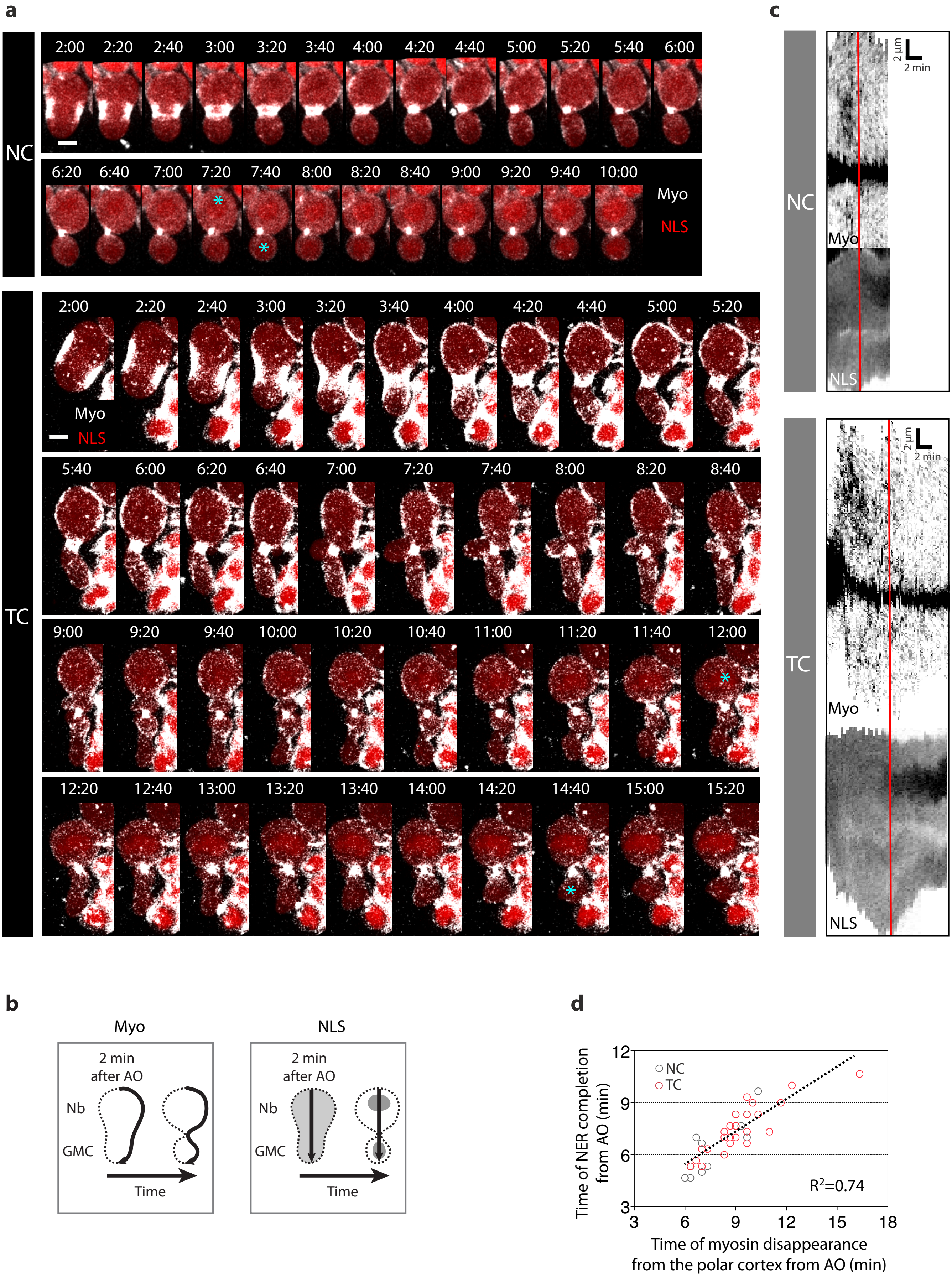
a
NC
2:00 2:20 2:40 3:00 3:20 3:40 4:00 4:20 4:40 5:00 5:20 5:40 6:00
6:20 6:40 7:00 7:20 7:40 8:00 8:20 8:40 9:00 9:20 9:40 10:00
Myo
NLS
TC
2:00 2:20 2:40 3:00 3:20 3:40 4:00 4:20 4:40 5:00 5:20
Myo
NLS
5:40 6:00 6:20 6:40 7:00 7:20 7:40 8:00 8:20 8:40
9:00 9:20 9:40 10:00 10:20 10:40 11:00 11:20 11:40 12:00
12:20 12:40 13:00 13:20 13:40 14:00 14:20 14:40 15:00 15:20
c
NC
2 µm
2 min
Myo
NLS
TC
2 µm
2 min
Myo
NLS
b
Myo
2 min after AO
Nb
GMC
Time
NLS
2 min after AO
Nb
GMC
Time
d
Time of NER completion from AO (min)
NC
TC
R²=0.74
12
9
6
3
3 6 9 12 15 18
Time of myosin disappearance from the polar cortex from AO (min)

Supplementary figure 6 | Myosin disassembly from the cortex correlates with the completion of nuclear envelope reassembly. (**a**) Time-lapse images of cells expressing GFP::NLS (NLS, red) and Sqh::RFP (Myo, grey) and carrying NC or TC. Time=min:sec. Time 0:00 corresponds to anaphase onset. Blue asterisks indicate the time of appearance of the GFP::NLS signal in the nuclei. Scale bars=5μm. (**b**) Scheme showing the method for making the kymographs to represent cortical myosin distribution (Myo, left scheme) and nuclear GFP::NLS distribution (NLS, right scheme) over time shown in c. (**c**) Kymographs of cortical myosin (Myo, top panel, grey) and NLS signals (NLS, bottom panel, grey) from one pole to the other over time for cells with NC or TC. The red vertical lines correspond to the time of myosin disappearance from the cortex, which correlates with the initiation of NLS nuclear accumulation. Note that myosin cortical localization is prolonged in cells with TC, which is associated with a delay in NLS nuclear accumulation. The X and Y axes scale bars correspond to 2 minutes and 2μm respectively. (**d**) Graph showing the linear correlation (R^2=0.74) between the time of myosin disappearance from the polar cortex and the time of GFP::NLS nuclear accumulation, which corresponds to NER completion.

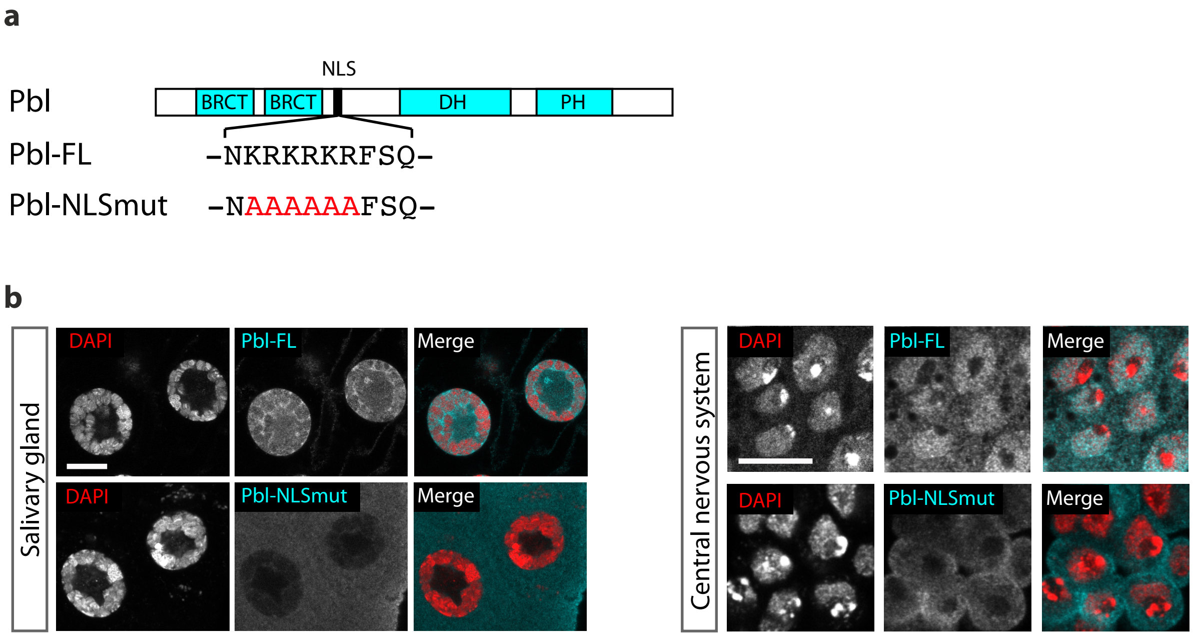

Supplementary figure 7 | Pbl accumulates in the nucleus at the end of mitosis via its NLS sequence. (**a**) Scheme showing the different domains of Pbl and the NLS sequence. The six alanines substitutions in the NLS sequence of Pbl-NLSmut are highlighted in red. (**b**) Images of fixed salivary gland cells and neuroblasts from *pbl* mutant third instar larvae expressing Pebble full length (Pbl-FL) or Pebble NLS mutant (Pbl-NLSmut) under its endogenous promoter, stained with anti-Pbl antibodies (grey in second panel and cyan in Merge) and DAPI (grey in first panel and red in Merge). In contrast to Pbl-FL, which brightly marks the nuclei in salivary gland and neuroblast cells, Pbl-NLSmut signal is mainly cytoplasmic. Scale Bars=10µm.

Supplementary Table 1

Name	Genotype	Fig.
-NC<br><br><br>-TC	-yw; P_{sqh}>sqh::GFP42/+; P_{H2Az}>H2Az::mRFP1/+<br>yw; P_{sqh}>sqh::RFP/+; $P_{ubi\text{-}p63E}$>GFP(S65T)::NLS (GFP::NLS in the text) /+<br>-yw; P_{sqh}>sqh::GFP42/+; P_{H2Az}>H2Az::mRFP1, P_{hsp70}>I-CreI/+<br>or yw, P_{hsp70}>I-CreI; P_{sqh}>sqh::GFP42/+; P_{H2Az}>H2Az::mRFP1/+<br>or yw, P_{hsp70}>I-CreI; P_{sqh}>sqh::RFP/+; $P_{ubi\text{-}p63E}$>GFP(S65T)::NLS /+	1; 2; 4b, c, e-i; S1 S4
	sqh^{AX3}; P_{sqh}>sqh::GFP42/+; P_{H2Az}>H2Az::mRFP1/+	3a-e; S3a
-NC<br>-TC	-yw; P_{sqh}>sqh::dendra2/+<br>-yw, P_{hsp70}>I-CreI; P_{sqh}>sqh:: dendra2/+	3f; S5
-*pbl* NC<br>-*pbl* TC	-yw; P_{sqh}>sqh::GFP42/+; pbl^{MS}, P_{H2Az}>H2Az::mRFP1/pbl^{5}<br>-yw; P_{sqh}>sqh::GFP42/+; pbl^{MS}, P_{H2Az}>H2Az::mRFP1, P_{hsp70}>I-CreI/pbl^{5}	4; 6f
-NC<br>-TC	-P_{Nup107}>GFP::Nup107; +; P_{H2Az}>H2Az::mRFP1/+<br>-P_{Nup107}>GFP::Nup107; +; P_{H2Az}>H2Az::mRFP1, P_{hsp70}>I-CreI/+	5a-c
-NC<br><br>-TC	-yw; +; $P_{ubi\text{-}p63E}$>GFP(S65T)::NLS (GFP::NLS in the text) /P_{H2Az}>H2Az::mRFP1<br>-yw; +; $P_{ubi\text{-}p63E}$>GFP(S65T)::NLS (GFP::NLS in the text)/P_{H2Az}>H2Az::mRFP1, P_{hsp70}>I-CreI	5d-f
-Pbl-FL NC<br><br>-Pbl-FL TC<br><br>-Pbl-NLSmut	-yw; P_{sqh}>sqh::GFP42/+; pbl^{MS}, P_{H2Az}>H2Az::mRFP1/pbl^{3}, P_{pbl}>pbl-FL(86F8)<br>-yw, P_{hsp70}>I-CreI; P_{sqh}>sqh::GFP42/+; pbl^{MS}, P_{H2Az}>H2Az::mRFP1/pbl^{3}, P_{pbl}>pbl-FL(86F8b)<br>-yw; P_{sqh}>sqh::GFP42/+; pbl^{MS}, P_{H2Az}>H2Az::mRFP1/pbl^{3},	6

NC<br>- Pbl-<br>NLSmut TC	*P_{pbl}>pblNLSmut(86F8b)*<br>*-yw, P_{hsp70}>I-CreI; P_{sqh}>sqh::GFP42/+; pbl^{MS},*<br>*P_{H2Az}>H2Az::mRFP1/pbl^{3}, P_{pbl}>pbl-NLSmut(86F8b)*	
	w; C(2)EN; P_{sqh}>sqh::GFP40/+	S2
-Embryonic<br>-Epidermal<br>and pI SOP	*-sqh^{AX3}; P_{sqh}>sqh::GFP42/+*<br>*-w; P_{sqh}>sqh::GFP42/+; P_{UAS}> H2A::RFP, P{Gal4}$neur^{P72}$/+*	S3b
-NC<br>-TC	*-yw; P_{sqh}>sqh::RFP/+; $P_{ubi\text{-}p63E}$>GFP(S65T)::NLS /+*<br>*-yw, P_{hsp70}>I-CreI; P_{sqh}>sqh::RFP/+; $P_{ubi\text{-}p63E}$>GFP(S65T)::NLS /+*	S6
-Pbl-FL<br>-Pbl-NLSmut	*-yw; +; pbl^{3}, P_{pbl}>pbl-FL(86F8b)/pbl^{3}*<br>*-yw; +; pbl^{3}, P_{pbl}>pbl-NLSmut(86F8b)/pbl^{3}*	S7b
